# Parental Selenium Nutrition Affects the One-Carbon Metabolism and the Hepatic DNA Methylation Pattern of Rainbow Trout (*Oncorhynchus mykiss*) in the Progeny

**DOI:** 10.3390/life10080121

**Published:** 2020-07-25

**Authors:** Pauline Wischhusen, Takaya Saito, Cécile Heraud, Sadasivam J. Kaushik, Benoit Fauconneau, Philip Antony Jesu Prabhu, Stéphanie Fontagné-Dicharry, Kaja H. Skjærven

**Affiliations:** 1 INRAE, University of Pau and Pays de l’Adour, E2S UPPA, NUMEA, 64310 Saint Pee sur Nivelle, France; pauline.wischhusen@inrae.fr (P.W.); cecile.heraud@inrae.fr (C.H.); sadasivam.kaushik@inrae.fr (S.J.K.); benoit.fauconneau@inrae.fr (B.F.); 2 Institute of Marine Research, P.O. Box 1870, 5020 Bergen, Norway; takaya.saito@hi.no (T.S.); antony.philip@hi.no (P.A.J.P.); kaja.skjaerven@hi.no (K.H.S.)

**Keywords:** selenium, methionine cycle, transsulfuration, nutritional programming, DNA methylation, rainbow trout

## Abstract

Selenium is an essential micronutrient and its metabolism is closely linked to the methionine cycle and transsulfuration pathway. The present study evaluated the effect of two different selenium supplements in the diet of rainbow trout (*Onchorhynchus mykiss*) broodstock on the one-carbon metabolism and the hepatic DNA methylation pattern in the progeny. Offspring of three parental groups of rainbow trout, fed either a control diet (NC, basal Se level: 0.3 mg/kg) or a diet supplemented with sodium selenite (SS, 0.8 mg Se/kg) or hydroxy-selenomethionine (SO, 0.7 mg Se/kg), were collected at swim-up fry stage. Our findings suggest that parental selenium nutrition impacted the methionine cycle with lower free methionine and S-adenosylmethionine (SAM) and higher methionine synthase (*mtr*) mRNA levels in both selenium-supplemented treatments. DNA methylation profiling by reduced representation bisulfite sequencing (RRBS) identified differentially methylated cytosines (DMCs) in offspring livers. These DMCs were related to 6535 differentially methylated genes in SS:NC, 6890 in SO:NC and 7428 in SO:SS, respectively. Genes with the highest methylation difference relate, among others, to the neuronal or signal transmitting and immune system which represent potential targets for future studies.

## 1. Introduction

Selenium (Se) is an essential micronutrient in humans and animals, with selenoproteins exerting various metabolic functions [1]. Among vertebrates, fishes have been described to have a well-developed selenoproteome [2], but there is concern within the aquaculture sector with present feed formulations. The ongoing replacement of Se-rich fishmeal with plant protein sources [3,4] is associated with a decrease in dietary Se level provided to farmed fish reared over a long period [5,6]. Many of the characterized selenoproteins are known to influence antioxidant metabolism, but knowledge on the effects of dietary Se on other metabolic pathways is not well characterized [7].

As shown *in vivo*, in the case of a Se deficiency, an increase in glutathione levels possibly relates to a feedback mechanism by changes in redox state [8,9]. The major source for glutathione is cysteine, which is synthesized from homocysteine via the transsulfuration pathway [10]. On the other hand, Se deficiency can impair the transsulfuration pathway with decreased levels of cysteine, cystathionine and homocysteine [11,12]. Homocysteine is a key metabolite in the methionine cycle, which links the antioxidant system to one-carbon (1C) metabolism [13]. Studies in mice have confirmed that Se affects methionine metabolism with decreased betaine homocysteine methyltransferase activity and S-adenosylhomocysteine (SAH) levels [14,15,16]. Comparable studies in fish are lacking.

In the methionine cycle, methionine is activated by S-adenosylmethionine synthetase to form S-adenosylmethionine (SAM). In the cell, SAM is the universal donor for methylation reactions forming SAH. DNA methylation is a major regulatory mechanism for epigenetic modifications [17]. DNA methylation at repeated cytosine phosphate guanine (CpG) residues, especially when localized at the promoter region, is considered to influence gene expression [18]. Dietary supplementation of Se has been associated with both hyper- and hypomethylation in mice, but the relationship between Se and epigenetic mechanisms is still not fully understood [19]. The present work therefore aims to study the effect of parental Se nutrition in rainbow trout (*Oncorhynchus mykiss*) on the 1C metabolism and the hepatic DNA methylation pattern of the progeny. 

The period of embryonic development is extremely sensitive to environmental-induced epigenetic modifications. For example, the allocation of maternal gene products and nutrients to the yolk has been associated with regulation of key embryonic developmental processes and persisting changes in the phenotype of the progeny [20,21]. In zebrafish (*Danio rerio*), dietary inclusion of methyl group donors did not lead to changes in hatching rate or survival, but mRNA sequencing of the embryos revealed “hidden” effects of parental nutrition [22] which led to phenotypic changes at later life stages [23]. In rainbow trout, the maternal Se nutrition during oogenesis increased not only the number of spawning females, but also the Se levels in the oocytes, especially when provided in the form of organic Se [6]. Changing Se levels in the progeny during embryonic development were also associated with modifications in the oxidative status. 

In fish diets, Se supplementation becomes increasingly important to make up for the low Se levels detected in diets based on plant protein sources [24]. With regard to Se supplements, in addition to the widespread use of sodium selenite in terrestrial livestock nutrition, selenomethionine is the naturally dominant dietary seleno-compound, known to be a highly bioavailable form of Se also in mineral premixes [25]. These seleno-compounds, however, might exert different impacts on the 1C metabolism as they are metabolized through different routes [26]. Seleno amino acids are metabolized interchangeably with their sulfur analogues making selenomethionine to follow the methionine cycle, while inorganic Se compounds such as sodium selenite can be directly reduced to selenide to be incorporated into selenoproteins as selenocysteine [24].

In this context, the present study aims to make a comparison between the use of sodium selenite and hydroxy-selenomethionine (OH-SeMet), a pure form of the hydroxy-analogue of selenomethionine, as dietary supplements in plant protein-rich feeds for rainbow trout broodstock on the 1C metabolism and the hepatic DNA methylation pattern of the progeny.

## 2. Results

### 2.1. Parental Selenium Affects Transsulfuration Metabolites in Swim-up Fry

A decrease in cysteine and cysteinyl-glycine was detected in liver of female broodstock only when fed sodium selenite (SS) compared to the non-supplemented control (NC). In addition, homocysteine levels were higher in fish fed OH-SeMet (SO) compared to the two other groups. No effect of the dietary Se on hepatic aminothiol concentrations was detected in males, which had generally lower hepatic aminothiol levels compared to females (Table 1).

A parental effect of Se in swim-up fry could be detected for cysteine as well as cysteinyl-glycine, which were both significantly lower in fry originating from parents fed Se-supplemented diets compared to the NC group (Table 2). This was accompanied by a decrease in pyridoxamine levels, but other B vitamins (folate and vitamin B12) including the pyridoxamine derivate pyridoxal were not significantly affected. Cystathionine, glutathione and γ-glutamyl-cysteine levels were not significantly different between the Se treatments. Similarly, parental Se treatment had no significant effect on the homocysteine level detected in swim-up fry.

### 2.2. Parental Selenium Nutrition Affects the Methionine Metabolism in Swim-up Fry

**Figure 1:**
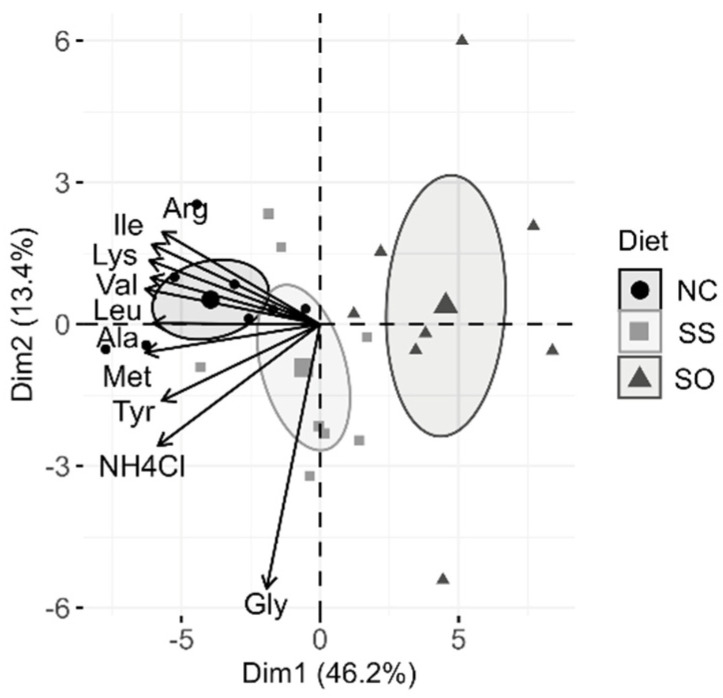
PCA biplot of free amino acids and related compounds measured in whole-body swim-up fry. Arrows represent the 10 most contributing variables to the model. Ellipses represent the 95% confidence intervals around a center of eight pooled samples per dietary treatment.

In the whole body of swim-up fry, the methionine concentration was significantly decreased, when parents received Se-supplemented diets compared to fry from the NC group, with the lowest concentration observed in fries from SO treatment (Table 1). The decreased methionine levels were accompanied by a general decrease in both essential and non-essential amino acids. PCA analysis of free amino acids and N-metabolites in fry revealed a strong clustering of the data according to the three parental groups dominated by essential amino acids, with the main contributing variables being lysine, isoleucine, valine, leucine, methionine, threonine and histidine besides glutamine, ammonium chloride and glycine (Figure 1). The only amino acid that was significantly higher in Se-supplemented treatments compared to the control was asparagine, with a 18 ± 4 µg/mg sample in NC vs. a 34 ± 3 µg/mg sample in SS and a 44 ± 5 µg/mg sample in SO.

**Figure 2:**
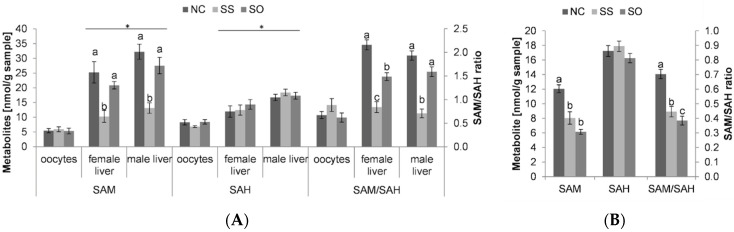
(**A**) SAM, SAH and the SAM/SAH ratio in whole-body swim-up fry; (**B**) SAM, SAH and the SAM/SAH ratio in broodstock tissue. Bars are the mean ± SEM (n = 8 in swim-up fry and female tissues and n = 5 in males). Means not sharing a common superscript letter are significantly different (*p* < 0.05) according to one-way ANOVA followed by Tukey’s HSD.

In broodstock liver tissue, a reduction in the SAM/SAH ratio was detected for the Se-supplemented groups (Figure 2A). SAM levels in males and females were, however, strongly affected by inorganic Se, without a significant difference between NC and SO in males that showed higher SAM as well as SAH levels compared to females. In oocytes, no significant difference in SAM or SAH levels and the SAM/SAH ratio was detected between treatments.

In the whole body of swim-up fry, the SAM/SAH ratio was low in both Se-supplemented treatments compared to the control with the lowest SAM/SAH ratio detected in the SO group (Figure 2B). The decrease in the SAM/SAH ratio can be related to the comparatively lower SAM levels observed in this group.

### 2.3. Parental Selenium Affects mRNA Levels of Genes Related to the One-Carbon Metabolism in Swim-up Fry

**Figure 3:**
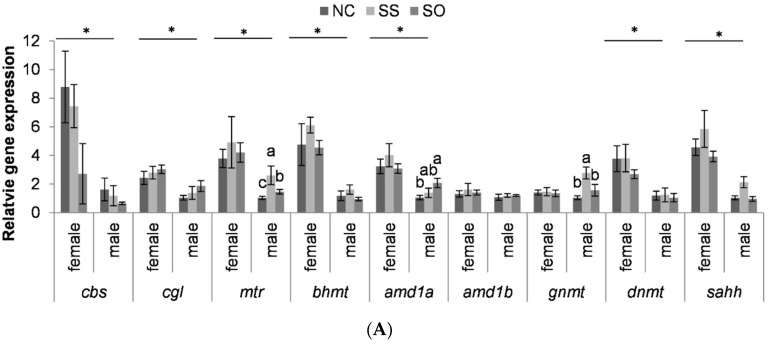

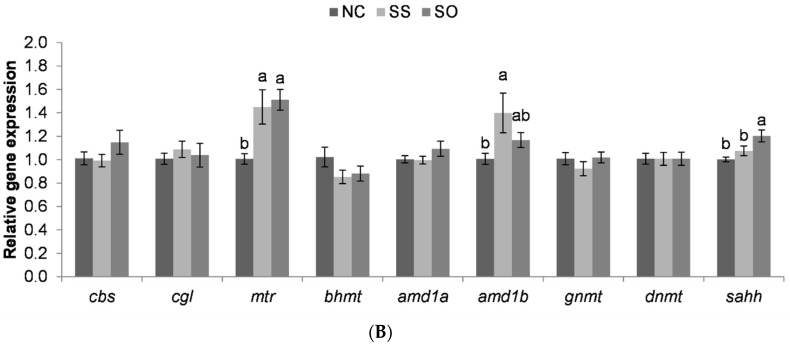
(**A**) Relative mRNA levels in whole-body swim-up fry from rainbow trout subjected to different Se treatments; (**B**) relative mRNA levels in parental liver tissue from rainbow trout subjected to different Se treatments. Data are normalized to β-actin and expressed as fold changes compared with the control group NC. In A, values are expressed relative to NC males. Bars are the mean ± SEM (A: n = 8; B n = 8 in female liver and n = 5 in male liver). Means not sharing a common superscript letter are significantly different (*p* < 0.05) according to one-way ANOVA followed by Tukey’s HSD.

Gene expression levels in female liver tissue were not significantly different between groups. Methionine synthase (*mtr*) expression was higher in male liver tissue when the fish were fed Se-supplemented diets, with the highest expression in the SS treatment (Figure 3A). In addition, in male liver tissue, the expression of adenosylmethionine decarboxylase 1a (*amd1a*) was higher in SO compared to NC and that of glycine N-methyltransferase (*gnmt*) in SS compared to the other two dietary treatments. Except for adenosylmethionine decarboxylase 1b (*amd1b*) and *gnmt*, the expression of the 1C metabolism-related genes analyzed was higher in the females than in the males.

Parental feeding of both SS and SO increased *mtr* gene expression in the swim-up fry compared to NC feeding (Figure 3B). In addition, the expression of *amd1b* was higher in SS compared to NC and that of adenosylhomocysteinase (*sahh*) was higher in SO compared to the two other groups.

### 2.4. Parental Selenium Resulted in a Weak Group-Wise DNA Methylation Clustering

**Figure 4:**
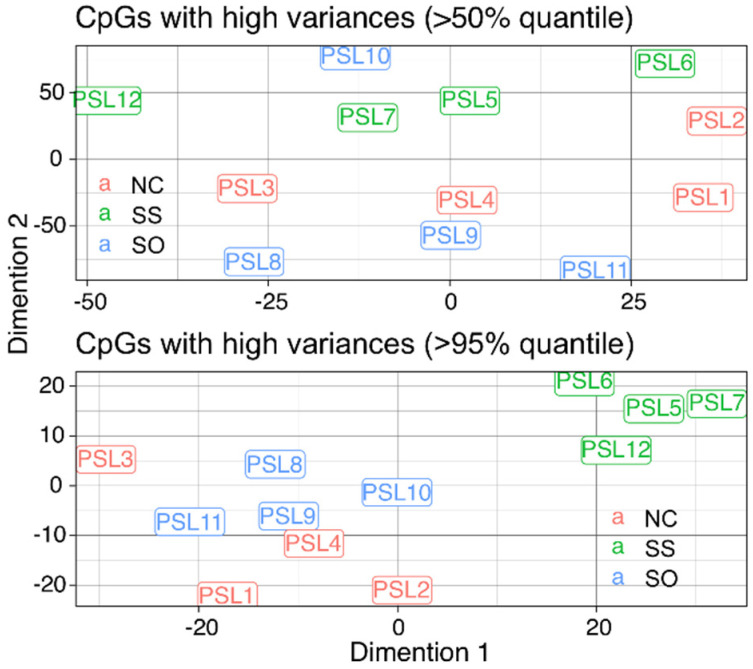
t-SNE analysis with the CpG sites using either the > 50% or > 95% quantile.

**Figure A1:**
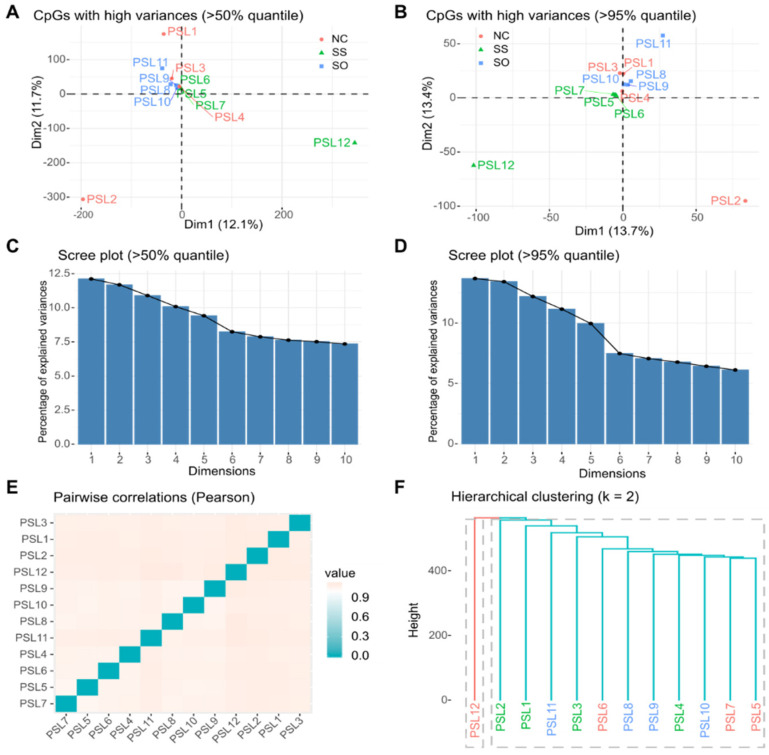
Clustering of the 12 RRBS samples by different algorithms. (**A + B**) PCA biplot by dietary treatment. (**C + D**) Scree plot with percentage of explained variance within the top 10 dimensions of the PCA. (**E**) Heatmap with sample–sample distance calculated by normalized Pearson’s correlation coefficient in a range between 0 and 1, with *d = 0* as *r = 1* and d=1 as *r = –1*. (**F**) Dendrogram with hierarchical clustering.

Reduced representation bisulfite sequencing (RRBS) data were first processed and aligned to the rainbow trout genome (Table A1). For downstream analysis, only uniquely mapped reads (47.6 ± 1.4%) were used. Of the 12 samples sequenced (4 per dietary group), none appeared as an outlier. In the search for the global methylation pattern with all the mapped CpG sites, t-SNE (t-distribution stochastic neighbor embedding) was used. The individual methylation pattern was stronger compared to group-wise global patterns, with only a weak group-wise clustering identified when using the 95th percentile of the CpC variance (Figure 4). The stronger individual variation compared to group-wise clustering was confirmed using other methods including PCA, hierarchical clustering and correlation analysis (Figure A1). 

### 2.5. Data Alignment Gives a Balanced Hepatic Methylation Pattern between Groups

**Figure 5:**
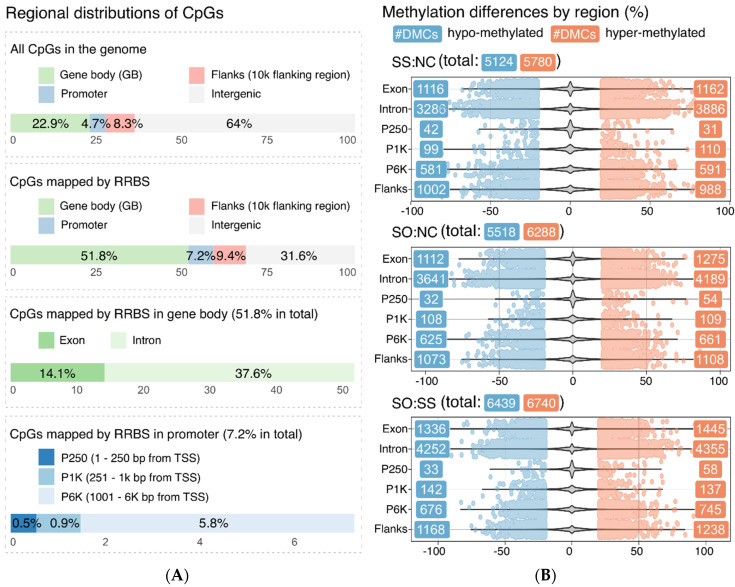
(**A**) Regional distributions of mapped/original CpG. (**B**) Regional distributions of methylation differences of the differentially methylated cytosines (DMC) with a 20% threshold. Three violin plots show the density of overall methylation differences for SS:NC, SO:NC, and SO:SS, with scattered dots indicating DMC.

The regional annotation showed that most of the mapped CpG originated with 51.8% from the gene bodies compared to the whole rainbow trout genome, where it accounts for 22.9% (Figure 5A). With 37.6%, most of the mapped CpG in the gene body were coming from the intron region. Further, promoters were more targeted by RRBS compared to the whole rainbow trout genome, with an increase from 4.7% to 7.2%.

The total number of differentially methylated cytosine (DMC) was comparable for the groups SS:NC (10904), SO:NC (11806) and SO:SS (13179) and, within each group, the number of hyper- and hypomethylated CpG sites was balanced even when divided into different sub-regions, exon and intron for the body and P250 for the proximal promoter, P1K for the promoter and P6K for the distal promoter region as well as flanks (Figure 5B). 

### 2.6. Parental Selenium Affects the DNA Methylation Pattern in Several Metabolic Pathways

**Figure 6:**
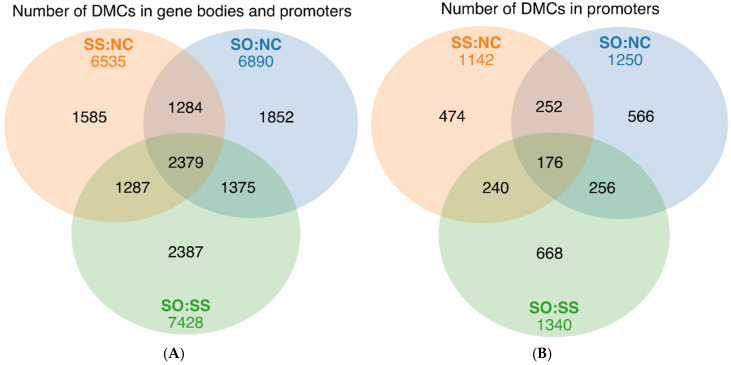
Venn diagrams summarizing the analysis of genes with different methylation patterns in NC vs. SS, NC vs. SO and SS vs. SO. (**A**) Genes are included with at least one DMC in gene body or promoter region; (**B**) Genes are included with at least one DMC in the promoter region.

Comparing NC with the SS treatment showed a total of 6535 differentially methylated genes (DMGs) from which 1142 DMGs had DMCs located in the promoter region (Figure 6). Similarly, in total, 6890 DMGs were detected between NC and the treatment receiving SO with 1250 DMGs showing DMCs located in the promoter region. The highest number of DMGs was detected between the two different Se-supplemented treatments with a total of 7428 genes of which 1340 DMGs had DMCs located in the promoter region. A synergetic effect of Se in NC vs. SS and NC vs. SO was detected on 3663 genes, whereas SS vs. SO displays a specific effect of the Se source on 2387 genes.

**Figure A2:**
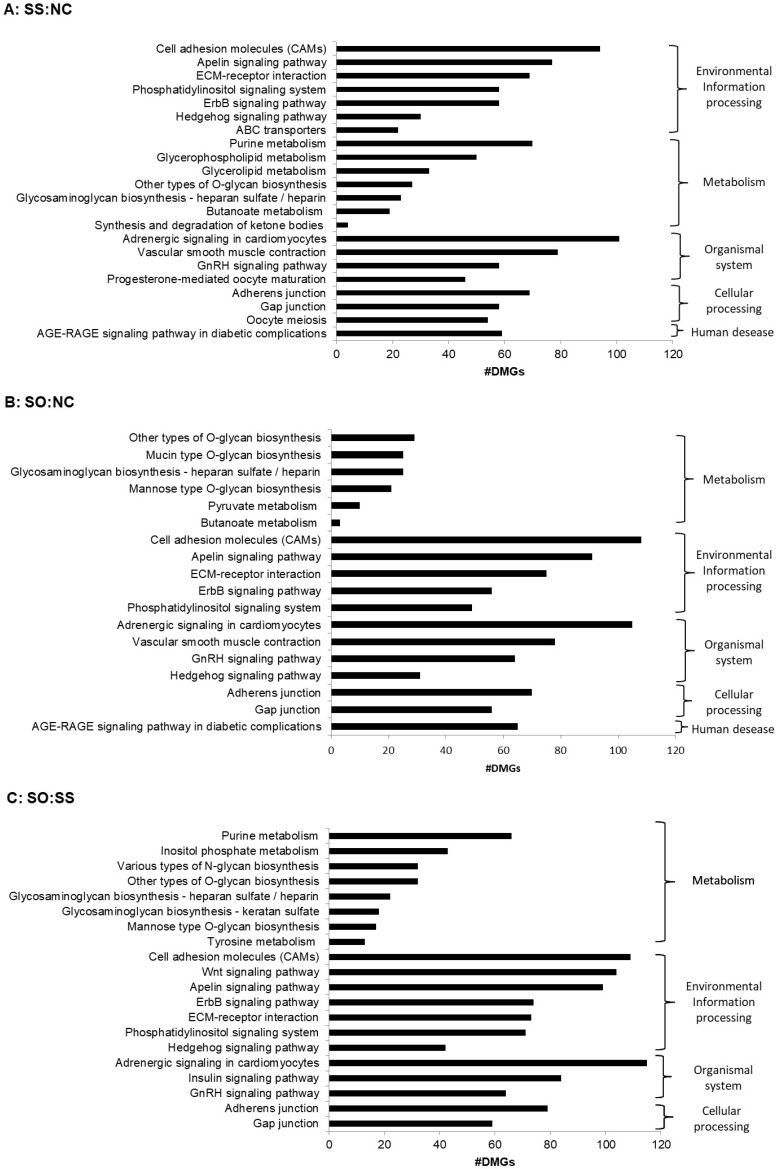
Enriched KEGG pathways in SS:NC (**A**), SO:NC (**B**) and SO:SS (**C**), with the number of differentially methylated genes (DMGs).

In all datasets, multiple KEGG pathways were significantly enriched—22 in SS:NC, 18 in SO:NC and 20 in SO:SS (Figure A2). These KEGG pathways relate to diverse biological mechanisms, mainly cellular metabolism and environmental information processing, but also the organismal system and cellular processing.

Among the five genes with the highest number of DMCs listed for each of the sub-regions (exon, intron, proximal promoter, promoter and distal promoter) in Table A2, Table A3 and Table A4, three genes were common. The limbic system-associated membrane, transcript variant X5 protein (*Isamp*) belongs to the immunoglobulin super-family, known to be expressed and excreted in the developing forebrain showed 15 DMCs in SS:NC, 24 in SO:NC and 22 in SO:SS, all located in the intron region. The methylation pattern revealed both hyper- and hypomethylated CpG sites. The DMCs of the other two genes were located in the promoter region. The radical S-adenosyl methionine domain containing protein 2-like (*viperin*) is a cytoplasmic antiviral protein that is induced by interferons. *Viperin* had five DMCs in SS:NC and three DMCs in SO:NC and SO:SS, respectively. In the inorganic Se treatment, the CpG sites were hypomethylated, but they were hypermethylated in the organic Se treatment. The third gene was gamma-aminobutyric acid receptor subunit rho-2 (*gabrr2*), which is an inhibitory neurotransmitter in the vertebrate brain. The DMCs of *gabrr2* were located in the distal promoter region and similar to *viperin* the gene was hypomethylated in SS:NC and hypermethylated in SO:NC.

### 2.7. Parental Selenium also Affects Methylation in Genes Related to the 1C Metabolism

Several genes related to the methionine cycle and transsulfuration pathway were identified, but mostly they contained only single DMC sites (Table 3). An effect on the genes that provide selenocysteine for the selenoprotein synthesis was only detected in the organic Se treatment. Nevertheless, selenoprotein I and selenoprotein U had DMCs in both Se-supplemented treatments. 

## 3. Discussion

### 3.1. Parental Selenium Nutrition and the Transsulfuration Pathway in the Progeny

**Figure 7:**
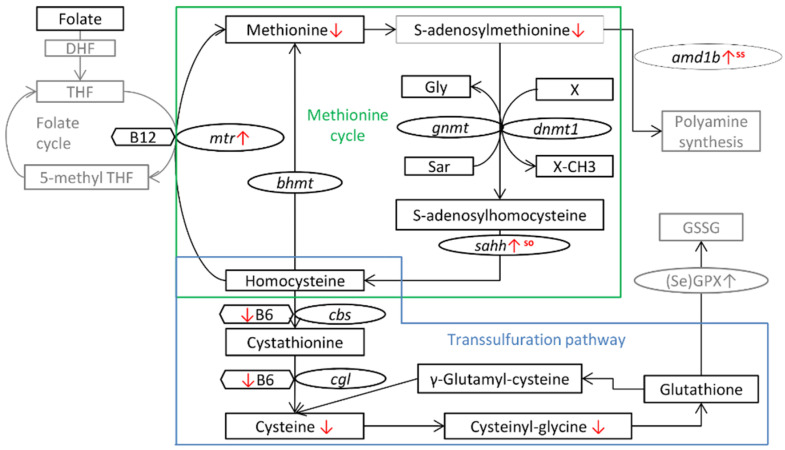
Effect of parental Se nutrition on the methionine cycle and transsulfuration pathway in the progeny of rainbow trout. Superscript indicates that the effect was only detected in the respective treatment.

Decreased levels of cysteine in rainbow trout fry originating from the parental group fed Se-supplemented diets are in contrast to reports in adult rats, mice and chicken, where rather an impaired transsulfuration with decreased cystathionine and cysteine levels has been described under dietary Se deficiency [12,14,15]. Decreased levels of cysteine were also detected in liver tissue of female broodstock of the sodium selenite treatment, indicating an effect independent of life stage in rainbow trout (Figure 7). In the present study, swim-up fry of the Se-supplemented treatments had lower pyridoxamine levels compared to the control group, which indicates an increased demand for vitamin B6 by parental Se nutrition. The combined effect of maternal Se and pyridoxal nutrition has been studied in porcine embryos by Dalto et al. [27,28], who described that the co-supplementation increased plasma seleno-dependent glutathione peroxidase levels in the progeny in the long term. In addition, the supply of organic Se and vitamin B6 stimulated the expression of elongation factors, biological processes related to translation and the mitotic cell cycle in five-day-old embryos [27]. Although the sulfur and seleno amino acids follow a similar pathway, the most important reaction for selenocysteine is its reduction to selenide via selenocysteine lyase and further by selenophosphate synthetase that donates Se to the Sec-tRNA for the selenoprotein synthesis [26]. Both these enzymes are vitamin B6 dependent, highlighting the importance of this vitamin in Se metabolism. This reaction is independent of dietary Se form as both inorganic and organic forms undergo the reduction to selenide [29]. The impact of dietary Se form as observed in the present study could be questioned as in an earlier study it was shown that even in the parental sodium selenite treatment more than 94% of the Se in oocytes was either selenocysteine or selenomethionine [6]. Nevertheless, the higher selenomethionine levels corresponding to the higher total Se levels in the organic Se treatment might contribute to changes in the methionine cycle, providing methyl groups and homocysteine/Se-homocysteine for the transsulfuration pathway. Thus, it can be inferred that the higher redox status of Se-homocysteine compared to homocysteine might favor transsulfuration, as its enzymes are readily regulated through the redox status [30].

### 3.2. Parental Selenium Nutrition and the Methionine Cycle in the Progeny

In the present study, parental Se had no effect on homocysteine levels in the swim-up fry, while studies in mice indicate an inverse correlation between liver Se and homocysteine levels [31,32]. An increased mRNA level of *mtr* in both Se-supplemented treatments could indicate that in rainbow trout, parental Se favors the re-methylation of homocysteine to methionine in the offspring. An increase in homocysteine can result in the accumulation of SAH, which is a competitive inhibitor of methyl-transferases and therefore associated to global hypomethylation [19]. In mammals, selenomethionine supplementation resulted in decreased hepatic SAH, but selenite on the contrary increased hepatic SAH [16,33]. In the present study, the difference was not significant. Nevertheless, increased mRNA levels of *sahh* indicate that SAH might be increasingly metabolized to homocysteine in the organic Se treatment (Figure 7). The decrease in the SAM/SAH ratio in swim-up fry of Se-supplemented treatments due to a decrease in SAM levels is possibly due to the lower methionine levels. In the OH-SeMet treatment, it cannot be excluded that a competition of selenomethionine on active transporters reduced the methionine uptake in the gut [34]. However, considering the small fraction that selenomethionine represents compared to dietary methionine levels in this study, it might be rather indicative of a higher methionine flux in the organic Se treatment. The sequence of Se-compounds in the metabolism creates an additional drain on methyl groups, as selenide and other highly reactive seleno-compounds can be spontaneously methylated [35]. This process is of importance for inorganic Se sources which do not follow the methionine cycle and are directly reduced to selenide [36]. This hypothesis is supported by our data on broodstock liver, where a significant decrease in SAM was only observed in the selenite treatment. This might be an explanation why Speckmann et al. [16] reported a high SAM/SAH ratio in response to selenomethionine supplementation using Se-deficient conditions in mice where no methylation of seleno-compounds for removal could be expected. Several other studies in mice using higher Se levels detected no effect of Se on the SAM/SAH ratio [14,33,37]. A depletion of the methyl donor SAM can result in decreased DNA methyltransferases (DNMT) activity [38]. If in human colon carcinoma cells, administration of selenite inhibits DNMT activity [39], *dnmt1* expression in the present study was not affected by Se, similar to what was reported on hepatic mRNA levels in mice [16].

### 3.3. Parental Selenium Nutrition Affects the DNA Methylation Pattern of Genes Related to Several Metabolic Pathways

Analyzed liver tissue of rainbow trout fry revealed that the DNA methylation patterns of several genes are sensitive to parental Se nutrition. Alterations in DNA methylation by Se have been reported in several murine studies, although with somewhat contradictory results, as Se could be associated with both hyper- and hypomethylation [16,33,37,40]. The methylation of DNA can possibly regulate the spatial-temporal expression pattern of genes driving towards the development of a specific phenotype [18]. Genes directly related to the sulfur and Se metabolism presented methylation differences according to parental Se nutrition. Therefore, epigenetic marks might relate to metabolic differences observed in the present as well as in an earlier study on the expression of genes involved in the glutathione and antioxidant metabolism in rainbow trout fry [6]. Although an expected enrichment of the glutathione pathway could not be detected, genes of the glutathione metabolism including glutathione synthetase and glutathione-s-transferase kappa 1 were detected as DMGs. It has been reported that in cancer cells, selenite supplementation reactivates the transcription of glutathione-s-transferase π, another member of the glutathione-s-transferase family by a hypermethylation of the promoter region [41]. Most studies with cancer cells generally report the methylation of selenoproteins like glutathione peroxidase 1 and 3, methionine sulfoxide reductase B1 and selenium binding protein 1 in the promoter region [42]. In the present study with rainbow trout, parental Se nutrition did not result in changing methylation pattern for these genes, contrary to selenoprotein I, a potential target of parental Se nutrition on the progeny. Selenoprotein I is a protein involved in the formation of the glycerophospholipid phosphatidylethanolamine [43], belonging to the glycophospholipid metabolism KEGG pathway that was enriched in the sodium selenite treatment. A silencing of the gene has been associated with impaired neural development as it is essential in the myelination process [44]. In general, several genes with high changes in DNA methylation like *gabrr2* were related to brain signaling pathways and neurotransmission. Under physiologically relevant conditions, Se nutrition has been associated with a neuroprotective role on γ-aminobutyric acidergic neurons [45]. It remains unclear whether the changes in DNA methylation of neuronal signaling genes as observed here in the hepatic tissue would be similarly detected in other organs such as the brain. DNA methylation works on a time and spatial dimension as genes gain tissue-dependent importance and are also activated and deactivated at different developmental stages [46,47]. High methylation differences were also identified for genes with a role in immune protection, including the antiviral protein *viperin* that showed several DMC sites in the promoter region. *Viperin* was up-regulated by supra-nutritional Se feeding in rainbow trout as well as Atlantic salmon (*Salmo salar*) in earlier studies [48,49]. This indicates that the impact of Se on the inflammatory response in fish might not be limited to direct feeding effects, but also be exerted through an epigenetic process. In this context, similar to other natural feed additives [50], Se might act as an immunostimulant, improving fish immunity in the long term.

## 4. Materials and Methods 

### 4.1. Experimental Set up

The experiment was conducted at the INRAE experimental fish farm in Lées-Athas, France. Fish maintenance and experimental procedures were conducted by trained personnel in compliance with the European Directive 2010/63/EU for the protection of animals used for scientific purposes and the French Decree no. 2013–118 for animal experimentation.

Three-year-old rainbow trout (*Oncorhynchus mykiss*) broodstock (initial mean weight: 1.1 ± 0.2 kg in females and 0.9 ± 0.3 kg in males) from the same genetic group produced at the INRAE facilities of Lées-Athas (permit no. A64-104-1) were individually tagged and divided into three groups consisting of 25 females and 15 males. The fish were reared under natural photoperiod, as previously described [6], over six months and fed the respective diets once daily to apparent satiation. At spawning, oocytes from eight females per group were fertilized with pooled sperm received from males of the same dietary treatment collected on the same day. Fertilized eggs from each female were reared separately in small trays until swim-up fry stage supplied with flow-through spring water at 8 ± 1 °C.

### 4.2. Experimental Diets

The diets were based on plant ingredients with an 8% fish oil inclusion and designed to differ only in their Se content (Table 4), as previously described [6]. The NC diet at a basal Se level of 0.3 mg/kg was not supplemented with Se. The SS diet was supplemented with sodium selenite to a target level of 0.6 mg/kg (analyzed concentration, 0.8 mg/kg) and the SO diet was supplemented to the same target level of 0.6 mg/kg with OH-SeMet (Selisseo®, Adisseo SAS, Antony, France), resulting in a final Se concentration of 0.7 mg/kg.

### 4.3. Sampling

The broodstock fish were anaesthetized with benzocaine for stripping and afterwards euthanized by a sharp blow to the head for liver dissection in both males and females. For each individual female, samples of pooled oocytes after stripping (1 g sized samples) and progeny at swim-up fry stage (whole-body fry) killed by an overdose of benzocaine were withdrawn. Moreover, a total of 36 individual swim-up fry livers were randomly dissected on the same day at the Ecology and Fish Population Biology facility in Saint-Pée-sur-Nivelle, France [52], originating from 12 females (n = 4 females per dietary treatment). The three individual livers per female were pooled into a single sample tube for DNA extraction. All collected samples were immediately frozen in liquid nitrogen and stored at −80 °C until further analysis.

### 4.4. Metabolite Analysis

In 0.1 g of pooled whole-body swim-up fry, free amino acids and other N-metabolites were analyzed using the Biochrome Analyzer and post column ninhydrin reaction following deproteinization, as previously described [53]. The aminothiols of the transsulfuration and glutathione pathway including homocysteine, cysteine, γ-glutamyl-cysteine, reduced glutathione and cysteinyl-glycine as well as SAM and SAH were measured by HPLC using one sample extract. First, 0.3 g of broodstock liver tissue, 1 g of pooled oocytes or 1 g whole-body swim-up fry were homogenized with an ultra-turrax in a 20 mM phosphate, 1 mM EDTA (pH = 6.4) buffer. After centrifugation (10,000 g, 15 min, 4 °C), deproteinization of the supernatant was performed using a 10% metaphosphoric acid solution. The protocol for aminothiol analysis was adapted from Toyooka and Imai [54]. Derivatization was performed by adding 62.5 µL AccQ·Fluor™ borate buffer (Waters, Guyancourt, France), 5 µL 1.55N NaOH and 4.5 mM ABD-F buffer to 25 µL sample aliquot for 20 min at 60 °C. After, the reaction was stopped by addition of 12.5 µL of 1N HCL and cooling at 4 °C for 15 min. Separation was performed using a AccQTaq^TM^ column at 40 °C using gradient elution: 0–2 min 97% A, 3% C; 20 min: 96% A, 4% C; 25 min: 20% B, 80% C; 30–35 min: 97% A, 3% C with (A) aqueous solution of AccQTag^TM^ Eluent A; (B) ultra-pure water and (C) methanol. Aminothiols were detected with fluorescence (excitation 385 nm, emission 515 nm). SAM/SAH measurement was adapted from She et al. [55] with separation on a Revolve C18 at 40 °C with the following gradient: 0–10 min 95% A, 5% B; 20 min 30% A, 70% B; 35–45 min 95% A, 5% B with (A) 20mM phosphate buffer with 8mM OSA (pH 2.7, TFA adjusted) and (B) methanol. In 0.1 g of pooled whole-body swim-up fry, pyridoxine, pyridoxal and pyridoxamine were measured by ultra-performance liquid chromatography (UPLC) [56] and vitamin B12 and total folate were analyzed microbiologically using *Lactobacillus delruceckii ssp. lactis* and *Lactobacillus rhamnosus*, respectively, as previously described [57]. 

### 4.5. RNA Extraction and RT-qPCR

The RNA was extracted and analyzed by quantitative RT-qPCR on 0.1 g samples of broodstock liver and a pool of three whole-body swim-up fry, as previously described [6]. The primer sequences are given in Table 5. 

### 4.6. Statistical Analysis on Metabolic Analysis and Gene Expression Data 

Results are given as the mean ± SEM. Statistical analysis was performed using statistical software R (R Core Team). All data were tested for normality and homogeneity. Gene expression data were rank transformed before further analysis. Principle component analysis (PCA) was performed on the free amino acid dataset in search for biological clusters and outliers (R: factoextra [58]). One-way ANOVA was used to identify differences between Se treatments or sex. Tukey’s HSD was used as a post hoc test in case a significant difference (*p* < 0.05) was detected.

### 4.7. DNA Extraction, RRBS Library Preparation and Sequencing

DNA extraction on swim-up fry livers was performed using a QIAGEN DNeasy Blood and Tissue Kit (cat. no. 69504), following the manufacturer’s instruction. DNA quantity was measured using Qubit fluorometric quantitation (Life Technologies, Carlsbad, California, USA), ensuring that the sample contained a minimum of 200 ng of DNA. The DNA extract was stored at −20 °C before DNA methylation was measured by reduced representation bisulfate sequencing (RRBS) performed at the Biomedical Sequencing Facility BSF in Vienna, Austria.

The RRBS library preparation was performed, as previously described [23], on 100 mg genomic DNA including DNA digestion (Msp1 20 U, 16h at 37 °C), enzymatic adapter ligation (T4 DNA Ligase rapid), quantification and pooling. Bisulfite conversion was performed using EZ DNA Methylation-Direct Kit D5020, Zymo Research, but conversion reagent was used at 0.9× concentration with incubation for 20 cycles of 1 min at 95 °C, 10 min at 60 °C and a desulphonation time of 30 min to increase the number of CpG nucleotides covered. Enrichment PCR was performed after AMPure XP clean up and library concentrations were quantified with the Qubit Fluorometric Quantitation system (Life Technologies) and size distribution by a Bioanalyzer High Sensitive DNA Kit (Agilent). Sequencing was performed on Illumina HiSeq 3000/4000 instruments. The data have been stored in SRA [59] under the accession number PRJNA629594.

### 4.8. Rainbow Trout Genome and Genomic Annotation

The reference genome data of rainbow trout (Omyk_1.0) were downloaded from the NCBI assembly site (https://www.ncbi.nlm.nih.gov/assembly/ GCF_002163495.1). 

For genes with multiple RefSeq sequences, only the longest sequence was kept after eliminating overlapped isoforms. All the CpG sites in the genome were identified and split into four regions—gene body (GB), promoter (P), flanking regions around mRNA (flanks), and intergenic. Gene body was further divided into two sub-regions, intron and exon, whereas promoter was also divided based on the distance from the transcriptional start site (TSS) as P250 (1 bp–250 bp), P1K (251 bp–1000 bp) and P6K (1001 bp–6000 bp). Flanks were defined as a combination of 4K upstream from the 5′ end of P6Ks (equivalently 6001–10,000 bp from TSS) and 10K downstream of the 3′ end of mRNA. All the regions outside of gene bodies, promoters and flanks were annotated as intergenic. Each CpG site was defined as a unique and non-redundant region or sub-region according to the precedence of exon > intron > P250 > P1K > P6K > flanks > intergenic.

### 4.9. RRBS Data Processing

Illumina2bam tools (1.17.3; https://github.com/wtsi-npg/illumina2bam) were used to de-multiplex pooled samples. SAMtools [60] was used to convert BAM files into FASTQ, before quality check by FastQC (Babraham Institute; https://www.babraham.ac.uk) and MultiQC [61]. Adapters and low-quality reads in the RRBS mode based on Cutadapt [62] were removed with Trim Galore! (Babraham Institute). Long reads were trimmed to 50 bp, and reads were selected by in-house python scripts to keep only those digested by MspI and TaqI.

Reads were aligned to the rainbow trout genome by Bismark [63] with Bowtie 1 [64]. Two Bismark tools, bismark_methylation_extractor and coverage2Cystosine, were used to retrieve methylation calls at CpG sites. Reads were filtered by methylKit tool [65] when either the number of reads was above 99.9th percentile or less than or equal to 10.

Cluster analysis was performed by Rtsne [66] for t-SNE [67], with perplexity = 2 and factoextra [58] for PCA, scree plot and hierarchical clustering with Ward’s method.

Prior to differential methylation calculation, the unite function of methylKit was used to form SS:NC and SO:NC with NC as control and SO:SS with SS as control. Methylation differences were calculated by methylKit for all the CpG sites with methylation calls as a percentage and *p*-values by logistic regression. The SLIM method [68] was used to calculate q-values. CpG sites with a q-value of < 0.01 and ≥ 20% methylation difference were defined as differentially methylated cytosines (DMCs). Genes with at least one DMC in the gene body or promoter region are considered to be differentially methylated genes (DMGs). 

In-house R and Python scripts were coordinated in a pipeline by using Snakemake [69].

### 4.10. Functional Annotation and Statistical Analysis of DMGs

To find Kyoto Encyclopedia of Genes and Genomes (KEGG) [70] orthologues that correspond to rainbow trout genes, the results of BLASTKoala, GhostKoala [71] and KEGG Automatic Annotation Server (KAAS) [72] were merged. The precedence of BLASTKoala > GhostKoala > KASS was applied when conflicting annotation occurred. A total of 22501 orthologues along with 168 KEGG pathways were identified. Over representation analysis (ORA) on KEGG pathways and Gene Ontology (GO) terms [73] was performed on DMGs by the R package clusterProfiler [74,75]. 

The Wilcoxon signed-rank test (Wilcox) was used to test the differences of methylation rates between two groups in a pair-wise manner for KEGG pathways. 

A bootstrap version of the Kolmogorov–Smirnov test (KS-boot; the number of iterations: 1000) was used to test the methylation differences that are associated with a KEGG pathway against the methylation differences of the whole CpG sites in a region. All three methods of enrichment analysis were performed for all the defined regions, and the *p*-values were adjusted by the Benjamini–Hochberg procedure.

## 5. Conclusions

Our results demonstrate that in rainbow trout, parental Se nutrition decreased transsulfuration and modified the methionine cycle, as summarized in Figure 7. A decrease in the methyl donor SAM was noticed in parental fish and their offspring by Se supplementation. In the offspring, significant changes in the DNA methylation pattern were identified, especially for genes related to signal transmission and immune function, by parental Se supplementation with organic and inorganic Se forms. It could be suspected that such epigenetic changes might persist during subsequent growth and development of the fish, leading to long-term molecular and metabolic alterations in the progeny, which deserves further investigation.

## Figures and Tables

**Table 1:** Aminothiol concentrations (µg/g sample) measured in liver and oocytes of rainbow trout (*Oncorhynchus mykiss*) broodstock fed diets containing different levels and source of Se.

		Homocysteine	Cysteine	Cysteinyl-Glycine	Glutathione	γ-Glutamyl-Cysteine
Oocyte	NC	0.3 ± 0.0	6.7 ± 1.0	3.7 ± 0.4	16 ± 1	1.0 ± 0.1
	SS	0.3 ± 0.0	6.7 ± 0.6	4.1 ± 0.5	17 ± 1	1.0 ± 0.1
	SO	0.2 ± 0.0	8.1 ± 1.1	3.1 ± 0.3	13 ± 1	1.0 ± 0.1
*p-value*		*0.21*	*0.48*	*0.35*	*0.07*	*0.48*
Female liver	NC	1.4 ± 0.1 ^b^	33 ± 4 ^a^	53 ± 4 ^a^	551 ± 32	30 ± 4
	SS	1.1 ± 0.1 ^b^	17 ± 2 ^b^	37 ± 3 ^b^	530 ± 32	23 ± 2
	SO	3.2 ± 0.3 ^a^	38 ± 3 ^a^	48 ± 3 ^ab^	526 ± 47	28 ± 3
*p-value*		***<0.01***	***<0.01***	***0.01***	*0.88*	*0.29*
Male liver	NC	0.7 ± 0.1	21 ± 3	26 ± 3	511 ± 64	21 ± 2
	SS	1.0 ± 0.2	24 ± 5	23 ± 3	300 ± 66	10 ± 2
	SO	1.1 ± 0.3	33 ± 8	27 ± 4	465 ± 30	16 ± 5
*p-value*		*0.49*	*0.33*	*0.80*	*0.06*	*0.48*
Average	Female	1.9 ± 0.2 ^a^	29 ± 2	46 ± 2 ^a^	536 ± 22 ^a^	27 ± 2 ^a^
	Male	0.9 ± 0.1 ^b^	26 ± 3	25 ± 2 ^b^	434 ± 39 ^b^	13 ± 2 ^b^
*p-value*		***<0.01***	*0.43*	***<0.01***	***0.02***	***<0.01***

Values are the mean ± SEM (n = 8 in female tissue and n = 5 in males). ^a,b^ Within-rows values not sharing a common superscript letter are significantly different (*p* < 0.05) according to one-way ANOVA followed by Tukey’s HSD.

**Table 2:** Free amino acid, aminothiol, and B vitamin composition of swim-up fry from broodstock fed the different diets.

Dietary Group	NC	SS	SO	*p*-Value
Essential amino acids ^1^	1972 ± 79 ^a^	1737 ± 54 ^b^	1400 ± 65 ^c^	**<0.01**
Non-essential amino acids ^1^	2415 ± 50 ^a^	2351 ± 41 ^a^	2109 ± 60 ^b^	**<0.01**
Methionine ^1^	99 ± 5 ^a^	83 ± 3 ^b^	56 ± 4 ^c^	**<0.01**
Homocysteine ^1^	1.2 ± 0.1	1.1 ± 0.1	1.2 ± 0.1	0.86
Cystathionine ^1^	9 ± 1	6 ± 1	7 ± 1	0.21
Cysteine ^1^	21 ± 1 ^a^	17 ± 1 ^b^	17 ± 0 ^b^	**0.01**
Cysteinyl-glycine ^1^	28 ± 1 ^a^	24 ± 1 ^b^	24 ± 1 ^b^	**0.02**
Glutathione ^1^	179 ± 7	159 ± 7	169 ± 13	0.35
γ-Glutamyl-cysteine ^1^	18 ± 1	17 ± 1	16 ± 1	0.25
Taurine ^1^	688 ± 17	751 ± 18	724 ± 16	0.05
Pyridoxamine ^2^	0.24 ± 0.01 ^a^	0.21 ± 0.02 ^b^	0.18 ± 0.01 ^b^	**0.01**
Pyridoxal ^2^	1.82 ± 0.08	1.65 ± 0.06	1.85 ± 0.10	0.17
Folate ^2^	0.36 ± 0.03	0.37 ± 0.02	0.28 ± 0.02	0.05
Cobalamine ^2^	0.04 ± 0.00	0.04 ± 0.00	0.03 ± 0.00	0.51

^1^ (µg/g sample); ^2^ (µg/mg sample). Values are the mean ± SEM (n = 8). ^a,b,c^ Within-rows values not sharing a common superscript letter are significantly different (*p* < 0.05) according to one-way ANOVA followed by Tukey’s HSD.

**Table 3:** Differentially methylated genes (DMGs) related to sulfur and selenium metabolism.

DMGs		Hyper-/Hypomethylated DMC		
Gene ID	Gene name	SS:NC	SO:NC	SO:SS
**Methionine Cycle**				
110530927	S-adenosylmethionine synthase	0/1		
110537066	S-adenosylmethionine synthase-like		0/1	0/2
110502651	S-adenosylmethionine synthase-like	1/0 ^P^	0/2	1/0
110529528	S-adenosylmethionine decarboxylase proenzyme-like		0/1	0/1
110538418	DNA (cytosine-5)-methyltransferase 1-like, transcript variant X1		0/1	
110505844	DNA (cytosine-5)-methyltransferase 3A-like		1/0	
110532515	DNA (cytosine-5)-methyltransferase 3A-like, transcript variant X2			1/0
110497603	DNA (cytosine-5)-methyltransferase 3A-like, transcript variant X6	1/0		
110492301	DNA (cytosine-5)-methyltransferase 3B-like, transcript variant X1			1/0
110500231	Putative adenosylhomocysteinase 3		0/1 ^P^	
110494352	S-adenosylhomocysteine hydrolase-like protein 1 transcript variant X1		1/0	
110490243	Adenosylhomocysteinase 3-like	2/1	4/2	0/1
110522167	Putative adenosylhomocysteinase 3, transcript variant X2			0/1
**Glutathione Metabolism**				
110521555	Glutamate—cysteine ligase regulatory subunit-like	0/1		
110502703	Glutamate—cysteine ligase catalytic subunit-like, transcript variant X2	0/1		
110532297	Glutathione synthetase		0/1 ^P^	
110522620	Glutathione-specific gamma-glutamylcyclotransferase 1-like		2/0	
110537206	Gamma-glutamyltransferase 5-like,transcript variant X1	0/1 ^P^	1/0 ^P^	2/0 ^P^
100305229	Glutathione S-transferase kappa 1, transcript variant X1			1/0 ^P^
110492369	Glucose-6-phosphate 1-dehydrogenase-like, transcript variant X2		2/0	0/1
100305228	Peroxiredoxin 6, transcript variant X2		1/0	
110532317	Spermidine synthase		0/1 ^P^	
110535309	5-oxoprolinase (ATP-hydrolyzing)		0/1	1/2
110501851	Isocitrate dehydrogenase [NADP] cytoplasmic-like	1/0 ^P^		0/1 ^P^
110520228	Isocitrate dehydrogenase [NADP] cytoplasmic-like		1/1	
**Selenoprotein Synthesis and Selenoproteins**				
110494778	Methionyl-tRna synthetase 1		0/1	0/1
110488988	Methionyl-tRNA synthetase 2, mitochondrial	1/0		
110500323	Sep (O-phosphoserine) tRNA:Sec (selenocysteine) tRNA synthase			1/0 ^P^
110512109	tRNA selenocysteine 1-associated protein 1-like		0/1	
110528137	Eukaryotic elongation factor, selenocysteine-tRNA specific		1/0	
110529243	Selenoprotein I	0/3	5/0	7/0
100499413	Selenoprotein U	0/1 ^P^	1/0 ^P^	1/0 ^P^
110532070	Selenoprotein K, transcript variant X1		0/1	
110497881	Selenoprotein O-like			1/0
110525853	Thioredoxin reductase 2			0/1

^P^ located at the promoter region.

**Table 4:** Dietary composition.

Diet	NC	SS	SO
*Ingredients*			
Plant meals ^1^	74	74	74
Crystalline amino acids and attractant mixture ^2^	3.14	3.14	3.14
Soybean lecithin^3^	2	2	2
Fish oil ^3^	8	8	8
Vegetable oils ^4^	8	8	8
Astaxanthin (µg/g diet) ^5^	40	40	40
Vitamin and mineral mixture without Se ^6^	4.82	4.82	4.82
Sodium selenite (µg/g diet) ^7^	–	**0.71**	–
Hydroxy-selenomethione (µg/g diet) ^7^	–	–	**0.75**
*Analytical composition*			
Dry matter (DM, %)	96	98	97
Crude protein (% DM)	49	50	50
Total lipid (% DM)	23	22	23
Gross energy (kJ/g DM)	25	25	25
Ash (% DM)	6	6	6
Phosphorus (% DM)	1.2	1.1	1.2
Selenium (mg/kg dry feed) ^8^	**0.3**	**0.8**	**0.7**

^1^ Plant meals (% diet): 20% wheat gluten (Roquette), 18% corn gluten meal (Inzo), 15% soybean protein concentrate Estril®75 (Sopropêche), 6% soybean meal (Sud-Ouest Aliment), 5% rapeseed meal 00 (Sud-Ouest Aliment), 5% white lupin meal Farilup 500 (Terrena), 3% dehulled pea meal Primatex (Sotexpro), 2% whole wheat (Sud-Ouest Aliment). ^2^ Crystalline amino acids and attractant mixture (% diet): 1.34% L-lysine, 0.3% DL-methionine, 0.5% glucosamine, 0.3% taurine, 0.3% betaine, 0.2% glycine, 0.2% alanine. ^3^ Soybean lecithin from Louis François and fish oil from Sopropêche. ^4^ Vegetable oils (% diet): 4% rapeseed oil, 2.4% linseed oil, 1.6% palm oil (Daudry). ^5^ Provided as Carophyll® pink (DSM). ^6^ Vitamin and mineral mixture without Se (per kg diet): retinol acetate, 55,000 IU; cholecalciferol, 2,500 IU; DL-α-tocopherol acetate, 50 IU; sodium menadione bisulfate, 10 mg; thiamin-HCl, 1 mg; riboflavin, 4 mg; niacin, 10 mg; D-calcium pantothenate, 20 mg; pyridoxine-HCl, 3 mg; D-biotin, 0.2 mg; folic acid, 1 mg; cyanocobalamin, 10 µg; L-ascorbyl-2-polyphosphate, 50 mg; myo-inositol, 0.3 g; choline, 1 g; CaHPO_4_·2H_2_O, 33 g; CaCo_3_, 2.15 g; Mg(OH)_2_, 1.24 g; KCl, 0.9 g; NaCl, 0.4 g; FeSO_4_·7H_2_O, 0.2 g; ZnSO_4_·7H_2_O, 40 mg; MnSO_4_·H_2_O, 30 mg; CuSO_4_·5H_2_O, 30 mg; NaF, 10 mg; KI, 0.4 mg; CoCl_2_·6H_2_O, 0.2 mg. All ingredients were diluted with α-cellulose. ^7^ Sodium selenite contained 42% Se (Sigma-Aldrich) and hydroxy-selenomethionine contained 40% Se provided as Selisseo® (Adisseo). ^8^ Total Se was determined using inductively coupled plasma mass spectrometry (ICP MS, Agilent series 7500cx) by Ultra-Trace Analysis Aquitaine (UT2A, Pau, France) according to Vacchina and Dumont [51], with a calculated uncertainty of 15 µg/kg and a limit of quantification of 3 µg/kg.

**Table 5:** Oligonucleotide primers used to assay mRNA levels by Fluidigm PCR.

Gene	Accession No.	Forward Primer	Reverse Primer	Amplification Size
*amd1a*	XM_021611778.1	ccgtaccatcccaaggtttga	tcctgcttgtcggtctttgt	87
*amd1b*	XM_021600287.1	cagccagattttcccaaacgg	gcatgctcgttctcccagaa	108
*bhmt*	FR908041.1	cagagaagcacggtaactgg	ttctttgtgctgcatcaggt	188
*cbs*	NM_001124686.1	ccacctcaggcaatacaggt	aacatccaccttctccatgc	107
*cgl*	EU315111.1	caccaaccccaccatgaaag	gcgctggaagtaggctgaca	118
*dnmt1*	XM_021557911.1	ttgccagaagaggagatgcc	cccaggtcagcttgccatta	152
*gnmt*	XM_021585680.1	ctcaagtacgcgctgaagga	cactctggtcccctttgaagt	187
*mtr*	XM_021576690.1	aatgcaggtctgcccaatac	ctgatgtgtgcaggagtcgt	137
*sahh*	XM_021609053.1	atcaaacgggccacagatgt	tcgtaccttccatggcagc	167
*β-actin*	AJ438158.1	gatgggccgaaagacagcta	tcgtcccgtggtgacgat	105

*amd1*, adenosylmethionine decarboxylase 1; *bhmt*, betaine-homocysteine S-methyltransferase 1; *cbs*, cystathionine beta-synthase; *cgl*, cystathionine gamma-lyase; *dnmt1*, DNA methyltransferase 1; *gnmt*, glycine N-methyltransferase; *mtr*, methionine synthase; *sahh*, adenosylhomocysteinase.

## Abbreviations

*amd*	Adenosylmethionine decarboxylase
*bhmt*	Betaine-homocysteine S-methyltransferase
*cbs*	Cystathionine beta-synthase
*cgl*	Cystathionine gamma-lyase
CpG	Cytosine phosphate guanine
DMCs	Differentially methylated cytosines
DMGs	Differentially methylated genes
DNMT	DNA methyltransferase
*gnmt*	Glycine N-methyltransferase
*mtr*	Methionine synthase
NC	Non-supplemented control treatment
OH-SeMet	Hydroxy-selenomethionine
RRBS	Representation bisulfite sequencing
SAH	S-adenosylhomocysteine
*sahh*	Adenosylhomocystenase
SAM	S-adenosylmethionine
SO	OH-SeMet treatment
SS	Sodium selenite treatment

## Appendix A

**Table A1:** Alignment statistics of 12 RRBS samples.

No	Name	Diet	Total Reads	Uniquely Mapped	(%)	Multi-Mapped	(%)	Non-Mapped	(%)
**1**	PSL1	NC	53 987 762	26 310 305	48.7	19 820 505	36.7	7 856 952	14.6
**2**	PSL2	NC	39 364 790	19 543 117	49.6	14 534 198	36.9	5 287 475	13.4
**3**	PSL3	NC	54 833 812	27 173 708	49.6	20 233 149	36.9	7 426 955	13.5
**4**	PSL4	NC	63 481 758	30 305 331	47.7	24 622 757	38.8	8 553 670	13.5
**5**	PSL5	SS	59 570 698	27 527 855	46.2	23 489 555	39.4	8 553 288	14.4
**6**	PSL6	SS	45 143 094	21 814 078	48.3	17 312 386	38.4	6 016 630	13.3
**7**	PSL7	SS	67 495 806	31 151 354	46.2	27 389 024	40.6	8 955 428	13.3
**8**	PSL8	SO	54 472 443	25 214 607	46.3	22 345 586	41.0	6 912 250	12.7
**9**	PSL9	SO	69 558 057	33 562 782	48.3	26 840 717	38.6	9 154 558	13.2
**10**	PSL10	SO	79 827 373	38 293 256	48.0	31 322 516	39.2	10 211 601	12.8
**11**	PSL11	SO	55 391 941	25 516 081	46.1	21 796 848	39.4	8 079 012	14.6
**12**	PSL12	SS	51 950 394	23 789 256	45.8	20 152 309	38.8	8 008 829	15.4

## Appendix C

**Table A2:** Top five DMGs in the dataset SS:NC, with the highest number of CpG in each sub-region.

	Gene Symbol	Gene ID	Gene Name	Total DMC	DMC in the Region	Hyper-/Hypomethylated CpG
Exon	LOC110496419	110496419	Glycoprotein endo-alpha-1,2-mannosidase-like protein	10	10	0/10
	LOC110503414	110503414	TCDD-inducible poly [ADP-ribose] polymerase-like	10	8	8/0
	LOC110497587	110497587	SAM and SH3 domain-containing protein 1-like, transcript variant X2	10	7	0/7
	LOC110520294	110520294	Von Willebrand factor C domain-containing protein 2-like, transcript variant X1	7	7	5/2
	LOC110529233	110529233	MAM domain—containing glycosylphosphatidylinositol anchor protein 2-like	6	5	5/0
Intron	CTNNA2	110534326	Catenin alpha-2	18	18	11/7
	alk	110506268	ALK receptor tyrosine kinase	17	17	13/4
	**lsamp**	**110507545**	**Limbic system-associated membrane protein, transcript variant X5**	**15**	**15**	**9/6**
	NRXN2-like	110531840	Neurexin-2-like	16	12	5/7
	CDH4-like	110532012	Cadherin-4-like	13	12	5/7
P250	**LOC110498119**	**110498119**	**Radical S-adenosyl methionine domain-containing protein 2-like**	**5**	**5**	**0/5**
	LOC110493563	110493563	Septin-9-like	4	2	0/2
	COX6A2	100335037	Cytochrome c oxidase subunit VIa	3	2	0/2
	TIMP2-like	110487076	Metalloproteinase inhibitor 2-like	2	2	0/2
	LOC110490026	110490026	Mitogen-activated protein kinase kinase kinase 8-like	2	2	2/0
P1K	LOC110534101	110534101	Matrin-3-like	4	4	4/0
	LOC110489756	110489756	Transmembrane protein 14C-like	4	3	0/3
	LOC110486159	110486159	Proline-rich protein 15-like protein A	3	3	2/1
	TNFR11B-like	110506163	Tumor necrosis factor receptor superfamily member 11B-like	3	3	0/3
	TPPP3X2-like	110518569	Tubulin polymerization-promoting protein family Member 3-like, transcript variant X2	3	3	0/3
P6K	COX4I2-like	110492636	Cytochrome c oxidase subunit 4 isoform 2, mitochondrial-like	8	8	8/0
	LOC110523211	110523211	Oocyte zinc finger protein XlCOF6-like, transcript Variant X2	8	8	0/8
	LOC110498688	110498688	Fatty acid-binding protein, liver-type-like	7	7	7/0
	**LOC110505815**	**110505815**	**Gamma-aminobutyric acid receptor subunit rho-2-** **like**	**6**	**5**	**0/5**
	LOC110503024	110503024	Ras-related C3 botulinum toxin substrate 3-like	5	4	0/4

Genes in bold are commonly highly affected also in SO:NC and SO:SS.

**Table A3:** Top five DMGs in the dataset SO:NC, with the highest number of CpG in each sub-region.

	Gene Symbol	Gene ID	Gene Name	Total DMC	DMC in the Region	Hyper-/Hypomethylated CpG
Exon	LOC110490066	110490066	E3 ubiquitin-protein ligase rififylin-like, transcript variant X2	6	6	5/1
	LOC110520294	110520294	von Willebrand factor C domain-containing protein 2-like, transcript variant X1	6	6	3/3
	LOC110531157	110531157	Serine/threonine-protein kinase WNK2-like	7	5	0/5
	LOC110497587	110497587	SAM and SH3 domain-containing protein 1-like, transcript variant X2	6	5	1/4
	LOC110506316	110506316	Muscarinic acetylcholine receptor M4-like, transcript variant X1	6	5	0/5
Intron	**lsamp**	**110507545**	**Limbic system-associated membrane protein, transcript variant X5**	**24**	**24**	**9/15**
	LOC110505581	110505581	Placenta growth factor-like	15	15	9/6
	LOC110501635	110501635	Serine/threonine-protein kinase BRSK2-like	14	14	10/4
	LOC110506270	110506270	Protein kinase C-binding protein NELL1-like, transcript variant X1	13	13	5/8
	Catenin alpha-2	110534326	Catenin alpha-2	13	13	6/7
P250	LOC110501919	110501919	Alkyldihydroxyacetonephosphatesynthase,peroxisomal-like, transcript variant X2	5	3	2/1
	LOC110494831	110494831	Complement C1q-like protein 2	4	3	0/3
	LOC110508922	110508922	MARVEL domain-containing protein 2-like, transcript variant X2	4	3	3/0
	CRIP2-like	110505831	Cysteine-rich protein 2-like	3	3	3/0
	LOC110497531	110497531	Uncharacterized LOC110497531, transcript variant X2	4	2	2/0
P1K	LOC110493345	110493345	Gastrula zinc finger protein XlCGF17.1-like, transcript variant X1	4	4	4/0
	LOC110521247	110521247	Lactadherin-like, transcript variant X1	4	4	4/0
	LOC110533598	110533598	Ras-related protein Rab-24-like, transcript variant X1	4	4	0/4
	**LOC110498119**	**110498119**	**Radical S-adenosyl methionine domain-containing protein 2-like**	**3**	**3**	**3/0**
	NFATC3-like	110506757	Nuclear factor of activated T-cells, cytoplasmic 3-like	3	2	0/2
P6K	**LOC110505815**	**110505815**	**Gamma-aminobutyric acid receptor subunit rho-2-like**	**8**	**5**	**4/1**
	LOC110519930	110519930	Uncharacterized LOC110519930, transcript variant X2	6	5	0/5
	LOC110520086	110520086	Collagen alpha-1(XXVIII) chain-like	5	5	3/2
	taf6l	110531860	TATA-box binding protein associated Factor 6 like, transcript variant X1	5	5	5/0
	LOC110537362	110537362	Glutamate receptor 3, transcript variant X3	5	5	5/0

Genes in bold commonly highly affected also in SS:NC and SO:SS.

**Table A4:** Top five DMGs in the dataset SO:SS, with the highest number of CpG in each sub-region.

	Gene Symbol	Gene ID	Gene Name	Total DMC	DMC in the Region	Hyper-/Hypomethylated CpG
Exon	CDH2-like	110506386	Neural-cadherin-like	10	10	7/3
	PLEKHG7-like	110497700	Pleckstrin homology domain-Containing family G member 7-like	9	9	0/9
	NRXN2-like	110531840	Neurexin-2-like	21	8	2/6
	LOC110496419	110496419	Glycoprotein endo-alpha-1,2-mannosidase-like protein	8	8	8/0
	LOC110496815	110496815	Glutamate receptor ionotropic, kainate5-like	8	7	7/0
Intron	**lsamp**	**110507545**	**Limbic system-associated membrane protein, transcript variant X5**	**22**	**22**	**9/13**
	LOC110500600	110500600	Adhesion G protein-coupled receptor L3-like, transcript variant X5	23	20	17/3
	FBXL17X1	110525966	F-box and leucine rich repeat protein 17, transcript variant X2	18	16	13/3
	LOC110535694	110535694	Glutamate receptor ionotropic, delta-2, transcript variant X3	17	15	5/10
	ZNF407-like	110501552	Zinc finger protein 407-like	16	15	3/12
P250	LOC110488021	110488021	Calcitonin gene-related peptide type 1 receptor-like	8	7	7/5
	GATA 2-like	110494514	GATA-binding factor 2-like	7	6	6/0
	**LOC110498119**	**110498119**	**Radical S-adenosyl methionine domain-containing protein 2-like**	**4**	**3**	**3/0**
	LOC110508922	110508922	MARVEL domain-containing protein 2-like, transcript variant X2	3	3	3/0
	CRIP2-like	110505831	Cysteine-rich protein 2-like	4	2	2/0
P1K	LOC110527134	110527134	Methyltransferase-like protein 7A	5	5	0/5
	SEMA5A-like	110489566	Semaphorin-5A-like	6	4	0/4
	LOC110493345	110493345	Gastrula zinc finger protein XlCGF17.1-like, transcript variant X1	4	4	4/0
	LOC100135939	100135939	Proteoglycan 4, transcript variant X2	4	4	1/3
	GRTP1a-like	110527156	Growth hormone-regulated TBC Protein 1-A-like	4	4	0/4
P6K	LOC110523211	110523211	Oocyte zinc finger protein XlCOF6-like,transcript variant X2	8	8	8/0
	MARVELD2-like	110523471	MARVEL domain-containing protein 2-like	8	8	8/0
	MED12-like	110488993	Mediator of RNA polymerase II transcription subunit 12-like	9	7	7/0
	LOC110522593	110522593	F-box only protein 31-like, transcript variant X1	7	7	0/7
	**LOC110505815**	**110505815**	**Gamma-aminobutyric acid receptor subunit rho-2-like**	**6**	**6**	**6/0**

Genes in bold are commonly highly affected also in SS:NC and SO:NC.
